# White Matter Characteristics of Damage Along Fiber Tracts in Patients with Type 2 Diabetes Mellitus

**DOI:** 10.1007/s00062-022-01213-7

**Published:** 2022-09-16

**Authors:** Haoming Huang, Xiaomeng Ma, Xiaomei Yue, Shangyu Kang, Yifan Li, Yawen Rao, Yue Feng, Jinjian Wu, Wenjie Long, Yuna Chen, Wenjiao Lyu, Xin Tan, Shijun Qiu

**Affiliations:** 1 The First Clinical Medical College, Guangzhou University of Chinese Medicine, 510405 Guangzhou, Guangdong China; 2 Department of Radiology, The First Affiliated Hospital, Guangzhou University of Chinese Medicine, 510405 Guangzhou, Guangdong China; 3 Department of Radiology, Jingzhou First People’s Hospital of Hubei Province, 434000 Jingzhou, Hubei China; 4 Department of Radiology, The Affiliated Hospital (Nanfang Hospital), Southern Medical University, 510515 Guangzhou, Guangdong China; 5 Department of Geriatrics, The First Affiliated Hospital, Guangzhou University of Chinese Medicine, 510405 Guangzhou, Guangdong China; 6 Department of Endocrinology, The First Affiliated Hospital, Guangzhou University of Chinese Medicine, 510405 Guangzhou, Guangdong China

**Keywords:** Type 2 diabetes mellitus, Automatic fiber quantification, Tract-based spatial statistics, Diffusion tensor imaging, Neurite orientation dispersion and density imaging

## Abstract

**Purpose:**

The white matter (WM) of the brain of type 2 diabetes mellitus (T2DM) patients is susceptible to neurodegenerative processes, but the specific types and positions of microstructural lesions along the fiber tracts remain unclear.

**Methods:**

In this study 61 T2DM patients and 61 healthy controls were recruited and underwent diffusion spectrum imaging (DSI). The results were reconstructed with diffusion tensor imaging (DTI) and neurite orientation dispersion and density imaging (NODDI). WM microstructural abnormalities were identified using tract-based spatial statistics (TBSS). Pointwise WM tract differences were detected through automatic fiber quantification (AFQ). The relationships between WM tract abnormalities and clinical characteristics were explored with partial correlation analysis.

**Results:**

TBSS revealed widespread WM lesions in T2DM patients with decreased fractional anisotropy and axial diffusivity and an increased orientation dispersion index (ODI). The AFQ results showed microstructural abnormalities in T2DM patients in specific portions of the right superior longitudinal fasciculus (SLF), right arcuate fasciculus (ARC), left anterior thalamic radiation (ATR), and forceps major (FMA). In the right ARC of T2DM patients, an aberrant ODI was positively correlated with fasting insulin and insulin resistance, and an abnormal intracellular volume fraction was negatively correlated with fasting blood glucose. Additionally, negative associations were found between blood pressure and microstructural abnormalities in the right ARC, left ATR, and FMA in T2DM patients.

**Conclusion:**

Using AFQ, together with DTI and NODDI, various kinds of microstructural alterations in the right SLF, right ARC, left ATR, and FMA can be accurately identified and may be associated with insulin and glucose status and blood pressure in T2DM patients.

**Supplementary Information:**

The online version of this article (10.1007/s00062-022-01213-7) contains supplementary material, which is available to authorized users.

## Background

People with type 2 diabetes mellitus (T2DM) have a 1.5–2 times greater predisposition to neurodegenerative disorders than healthy individuals [1]. Studies have shown that T2DM accelerates brain aging and is associated with mild cognitive impairment and the development of Alzheimer’s disease [2]. In recent years, neuroimaging evidence has shown that T2DM is accompanied by microstructural abnormalities of the white matter (WM) in various areas of the brain, which are closely related to hyperglycemia and insulin resistance status [3–5].

The integrity of the WM is commonly analyzed with diffusion weighted imaging (DWI), and diffusion tensor imaging (DTI), one of the most frequently reported DWI reconstruction models, can be used to observe the changes in the WM microstructure in T2DM patients. By utilizing tract-based spatial statistics (TBSS) and tractography, researchers have reported alterations in the microstructural integrity of the white matter in T2DM patients, including a decrease in fractional anisotropy (FA), an increase in mean diffusivity (MD), and alterations in WM fiber tracts. These alterations have been demonstrated in pertinent regions including the cingulum (CGC), the superior longitudinal fasciculus (SLF) of the bilateral parietal lobes, and the uncinate fasciculus (UNF) of the right frontal lobe [4–7].

Due to the shortcomings of the Gaussian model assumption regarding water molecule movement, the DTI model faces some drawbacks in specifically identifying the microstructural characteristics in individual tissues [8]. The DTI-derived metrics are essentially nonspecific for different types of pathological changes in the fiber tracts [8]. The decrease in FA may be caused by a decrease in neurite density, and alterations in the dispersion of neurite orientation contribute to various microstructural changes [9]. The diffusion process in areas of low anisotropy and complex crossing fiber structures may also make it difficult to interpret the underlying pathology through DTI-derived metrics [10–12].

Notably, the neurite orientation dispersion and density imaging (NODDI) model based on multi-interval biophysics is a promising technique that can describe the density and orientation of the neurites that indicate the pathological changes in WM fiber tracts. Two NODDI-derived metrics, the intracellular volume fraction (ICVF) and the orientation dispersion index (ODI), represent neurite density and the dispersion of neurite orientation, both of which affect FA [13]. An in vivo study also showed that NODDI can identify potential nontissue sources contributing to the DTI findings, such as tau accumulation, and thus may provide greater specificity to pathology in neurodegenerative diseases [14]. In addition, to accurately resolving the ambiguity of crossing fibers and fit the NODDI model, various *q*-space sampling techniques were developed recently, among which the Cartesian grid imaging scheme (also known as diffusion spectrum imaging, DSI) has been shown to be capable of evaluating the T2DM-related microstructural integrity of the fiber tracts, such as those of the UNF and CGC, which are associated with patient cognitive status [4, 15].

Automatic fiber quantification (AFQ) is a new analytical method that uses deterministic tractography to reconstruct fiber bundles and estimate the pointwise diffusion parameters at 100 anatomically equivalent positions along the fiber trajectory for every specific bundle in which lesions on the fiber tracts can be located precisely at the individual level [16]. Due to the different shapes of long-range fiber bundles among subjects and the fact that the disease may invade local nodes in the fiber bundles, TBSS cannot comprehensively evaluate the damage to each fiber bundle of the nodes at the individual level [17]. AFQ overcomes the ability of TBSS to only evaluate the average diffusion change of the whole fiber bundle. Recently, AFQ has been used in the research of a variety of neuropsychiatric diseases [18, 19]. Nonetheless, the specific proportion of alterations and the associated pathology along the fiber tracts in T2DM remain unclear.

It is important to precisely characterize the brain microstructural alterations of the WM in T2DM patients to provide biomarkers for detecting brain microstructural damage in T2DM. In this study, we used TBSS and AFQ to analyze DSI data to comprehensively investigate WM damage in patients with T2DM. Moreover, DTI and NODDI models were utilized to detect the biophysical microstructural damage of the WM in T2DM patients.

## Methods

### Participants

The current study was approved by the local Medical Research Ethics Committee. A total of 61 T2DM patients younger than 60 years old (composing the T2DM group) were recruited, and another 61 healthy subjects composed the HC group. Written informed consent was signed by all participants before they began the trial. The HC subjects were age-matched and sex-matched volunteers who underwent a routine physical examination, did not have any history of blood glucose abnormalities, and tested normal on the fasting fingerprick blood sugar test (GA‑6 blood glucose meter; Sinocare Inc., Changsha, China) before the MRI scan. All the subjects were right-handed, Han Chinese adults and native Chinese speakers. In addition, the T2DM patients were monitored to ensure that their blood glucose levels were stably controlled. The diagnosis of T2DM was based on the American Diabetes Association guidelines: diabetes symptoms and hemoglobin A1c > 6.5% or A1c > 48 mmol/mol and/or a fasting plasma glucose level of > 7.0 mmol/L and/or a random plasma glucose level of > 11.1 mmol/L and/or a 2‑h glucose level of > 11.1 mmol/L after an oral glucose tolerance test [20].

For each registered participant, characteristics such as age, sex, education level, systolic blood pressure (SBP), diastolic blood pressure (DBP), and body mass index (BMI) were recorded as basic information. For T2DM subjects, the duration of diabetes, glycosylated hemoglobin A1c (HbA1c), fasting blood glucose (FBG) level, and fasting insulin (FINS) level were also documented. All subjects underwent the Montreal Cognitive Assessment (MoCA) and the Mini-Mental State Examination (MMSE) tests to assess their cognitive status. Individual insulin-resistant status was estimated by the homeostatic model assessment for insulin resistance (HOMA-IR), which was conducted with HOMA2 Calculator 2.2.3 (The Oxford Centre for Diabetes, Endocrinology and Metabolism, University of Oxford, UK). In addition, all the participants were examined in detail by professional neurologists to exclude those with positive neurological symptoms.

Subjects with the following characteristics were excluded from the trial: (1) unstable blood glucose control; (2) pregnancy; (3) organic lesions/abnormalities in the brain, such as infarction, hemorrhage, tumors, vascular malformation, trauma, brain surgery, or congenital defects; (4) previous history of neurological diseases or psychiatric disorders, such as depression, schizophrenia, epilepsy or Parkinson’s disease; (5) chronic infections or systemic diseases (e.g., autoimmune diseases or organ failure), a history of tumors, a history of alcohol dependence or substance abuse; (6) complications of diabetes (e.g., nephropathy, peripheral neuropathy, ketoacidosis, or diabetic-related retinopathy); (7) moderate to severe hypertension or hyperlipidemia; (8) contraindications to MRI examinations, such as metallic implants or claustrophobia; (9) other types of abnormal glucose conditions or diabetes (e.g., impaired glucose tolerance or type 1 diabetes mellitus); and (10) other factors that might affect thyroid function.

### Magnetic Resonance Image Protocols and Preprocessing

Neuroimages were acquired on a 3 T MRI scanner (MAGNETOM Prisma; Siemens Healthcare, Erlangen, Germany) equipped with a 64-channel head coil. Head motions were minimized with a soft sponge pad during the scan. T1-weighted imaging (T1W1), T2-weighted imaging, and T2-fluid-attenuated inversion recovery images were acquired to rule out other neurologic disorders, such as lacunar infarctions and moderate to severe WM disease [21]. DSI data were acquired by using a half-coverage Cartesian q‑space grid scheme with a radial grid size of 4 and 11 b‑values (b = 300, 350, 650, 950, 1000, 1350, 1650, 1700, 2000, 2700, and 3000 s/mm^2^) along 99 diffusion gradient directions were included in the acquisition. Two images were acquired at b = 0 s/mm^2^, and 100 slices of DSI data per subject were acquired. The detailed MRI scanning protocols used are shown in Table S1. The diffusion data were processed with the *topup* tool in FSL 6.2.1 (The Oxford Centre for Functional MRI of the Brain, University of Oxford, UK) for distortion correction. Head motion corrections were performed via a 3dshoreline strategy in the Qsiprep 0.14.2 (Lifespan Informatics & Neuroimaging Center, University of Pennsylvania, USA) package [22]. According to a previous study [23], quality control (QC) was performed via visual inspection and through a QC file, automatically generated during preprocessing that reported signal dropout (bad slices) of the subject’s DWI data. If more than 1 bad slice was reported, the data were excluded. For the convenience of downstream analyses, DSI data were skull stripped and nonlinearly registered to the subjects’ T1 spaces during preprocessing. DTI metrics such as FA, MD, axial diffusivity (AD), and radial diffusivity (RD) were then calculated with the *dtifit* command in FSL. The NODDI model was reconstructed with the AMICO (Department of Computer Science, University of Verona, Italy) toolkit before calculation of the ICVF and ODI [24].

### TBSS

TBSS was performed on diffusion metrics to identify WM abnormalities between patients and HCs. By following the TBSS protocol in FSL, FA images were slightly eroded to remove brain-edge artifacts and outliers. The preprocessed FA images were then registered to the FMRIB58-FA template provided by FSL and resampled to 1 × 1 × 1 mm in Montreal Neurological Institute (MNI) space. The mean FA was skeletonized with a threshold of 0.3 to create a binary FA skeleton mask. Finally, subject FAs along with other diffusion metrics were projected onto the FA skeleton.

### AFQ

To further quantify the tissue properties within major fiber tracts in an individual brain, an AFQ tractometry pipeline [16] was constructed using the pyAFQ 0.12 (Brain Development & Education Lab, Stanford University, USA) package in Python. Briefly, diffusion-derived maps were loaded into the pyAFQ API. Second, individual data were registered to the MNI T2 template. The waypoint and bundle probability maps of 18 major bundles, namely, the bilateral anterior thalamic radiation (ATR), CGC, corticospinal tract (CST), inferior fronto-occipital fasciculus (IFOF), inferior longitudinal fasciculus (ILF), SLF, arcuate fasciculus (ARC), UNF, and two fibers that cross the midline, the forceps minor (FMI) and forceps major (FMA). DWI data were resampled into the MNI space and registered to the subject space for bundle tracking. Using the DTI model and a deterministic fiber tracking algorithm, streamlines were identified between 10 mm and 1 m in length, with a step size of 0.5 mm; tracking stopped in voxels with FA values < 0.2; turning angles larger than 30 ° were excluded. Furthermore, streamlines 5 times greater than the standard deviations from the Mahalanobis distance or 4 times greater than the standard deviations from the mean length were excluded; each fiber group was resampled to 100 equidistant points to which diffusion metrics were projected. Finally, a visual inspection was performed to detect unusual tracking results. The batch processing was performed by setting up a “*.toml” configuration file and automatically completed through the pyAFQ command line tool.

### Statistical Analysis

Statistical tests were performed in R software 4.0 (R Core Team and R Foundation). For other demographic, clinical, and psychological assessment variables, if the data were normally distributed, Student’s t test was used to identify the significant differences between groups; otherwise, the Kruskal–Wallis test was used. Population-space WM skeleton mappings were subjected to FSL’s *randomise* program for voxelwise analyses between two groups using a nonparametric permutation test with 10,000 permutations and adjusting for age, sex, education, and BMI followed by threshold-free cluster enhancement of the *t* statistic map with familywise error (FWE) correction, and *P*_FWE_ < 0.05 was considered to be significant. Diffusion metrics across each fiber tract were reformatted to subject-level 100-point 1‑dimensional arrays. ANCOVA tests were performed on the mean diffusion metrics of each fiber tract with age, sex, education, and BMI as covariables, and a false discovery rate (FDR)-corrected *P*_FDR_ < 0.05 was considered to be significant. Group level pointwise analyses of diffusion measures were performed using *randomise* in FSL (5000 permutations) with a familywise error rate controlled at *P*_FWE_ < 0.05 to identify differences among groups after regressing out age, sex, education, and BMI. To further explore the association between the node-specific alterations of the fiber tracts and diabetes and cognition status, significantly different node diffusion metric values were extracted, and aberrant diffusion metric values of continuous aberrant nodes were averaged. Spearman partial correlation analysis was conducted on node metric values and diabetes duration, HbA1c, FBG, FINS, HOMA-IR, SBP, DBP, MoCA, and MMSE controlling for age, sex, education, and BMI. Given the exploratory nature of the correlation analysis, no *P*-adjusted method was applied.

## Results

### Participant Characteristics

The demographic, clinical, and cognitive measurements of 61 T2DM patients and 61 HC subjects are summarized in Table 1. There was no significant difference in age, sex, or education level between the participants in the two groups. Regarding the clinical testing and cognitive assessments, the SBP, DBP, BMI, MoCA scores, and MMSE scores were not significantly different between the groups (*P* > 0.05). All subjects (*n* = 122) passed the QC in the DSI data preprocessing.

**Table 1:** MRI scan sequence parameters

Sequence	TR/TE/TI (ms)	Slice thickness/gap (mm)	FOV (mm^2^)	Voxel size (mm^3^)	Acquisition time (min)
3D-T1WI	2530/2.98/–	1/0	256 × 256	1 × 1 × 1	5:58
T2WI	3650/92/–	5/1	220 × 220	0.7 × 0.7 × 5	0:49
T2-FLAIR	9000/84/2500	5/1	220 × 220	0.7 × 0.7 × 5	1:50
DSI^a^	4200/72/–	2/1	220 × 220	2 × 2 × 2	7:31

*T1W1* T1-weighted imaging, *T2WI* T2-weighted imaging, *T2-FLAIR* T2-fluid-attenuated inversion recovery, *DSI* diffusion spectrum imaging, *TR* repetition time, *TE* echo time, *TI* inversion time, *FOV* field of view

^a^DSI data were acquired by using a half-coverage Cartesian q‑space grid scheme with a radial grid size of 4. Eleven b‑values (b = 300, 350, 650, 950, 1000, 1350, 1650, 1700, 2000, 2700, and 3000 s/mm^2^) along 99 diffusion gradient directions were included in the acquisition, two b = 0 s/mm^2^ images were acquired, and one was taken in opposing phase-encoding directions

### TBSS

**Fig. 1:**
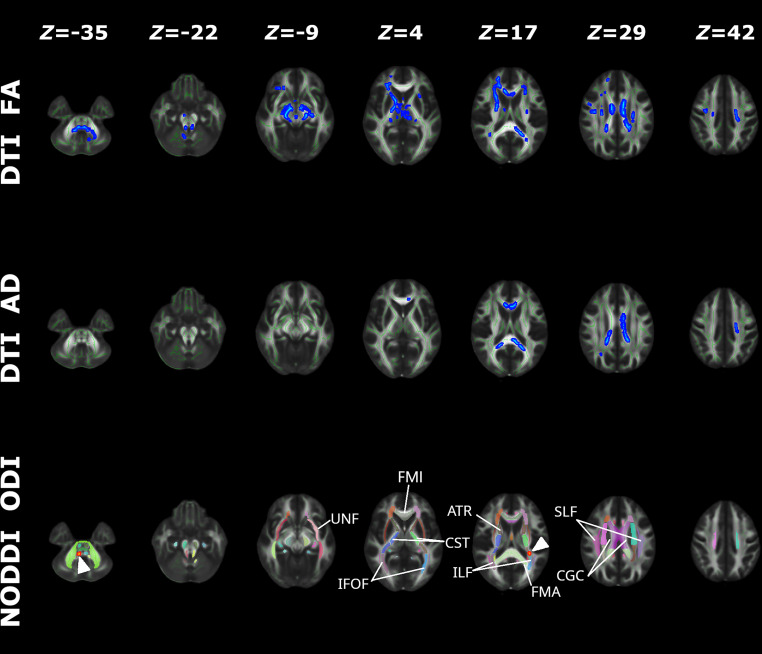
NODDI and DTI metric differences in the microstructure of the white matter. *Red* indicates significantly increased metrics in the T2DM group compared to the HC group with (1 − *P*_FWE_) > 0.95; *blue* indicates significantly decreased metrics in the T2DM group compared to the HC group with (1 − *P*_FWE_) > 0.95; *z* values indicate MNI coordinates of the cross-section slice; *arrowheads* highlight regions with abnormal ODI. The “*Z*” represents the MNI coordinate of the cross-section slice. *T2DM* type 2 diabetes mellitus, *HC* healthy controls, *FWE* familywise error, *DTI* diffusion tensor imaging, *NODDI* neurite orientation dispersion and density imaging, *FA* fractional anisotropy, *AD* axial diffusivity, *ODI* orientation dispersion index, *ATR* anterior thalamic radiation, *CGC* cingulum, *CST* corticospinal tract, *FMI* forceps minor, *FMA* forceps major, IFOF inferior fronto-occipital fasciculus, *ILF* inferior longitudinal fasciculus, *SLF* superior longitudinal fasciculus, *UNF* uncinate fasciculus

The main purpose of the TBSS analysis was to study the pattern of the altered microstructure of the WM as reflected by differences in the diffusion MRI (DTI and NODDI) metrics between participants with T2DM (*n* = 61) and those in the HC group (*n* = 61) (Fig. 1). The FA values were significantly lower in participants in the T2DM group than in the HC group in several WM regions: the ATR, corticospinal tract, IFOF, FMI, left UNF, and right SLF. The AD values were lower in the left corticospinal tract, FMI, and FMA. However, the right ATR and corticospinal tract and the left IFOF and ILF had significantly increased ODI values in patients with T2DM. Detailed cluster information about FA, AD, and ODI, including the total number of voxels, peak coordinates, *z* value, and anatomic locations, are listed in Tables S1, S2 and S3, respectively. No significant intergroup difference was found for the other diffusion metrics, such as MD, RD, and ICVF.

### AFQ

**Fig. 2:**
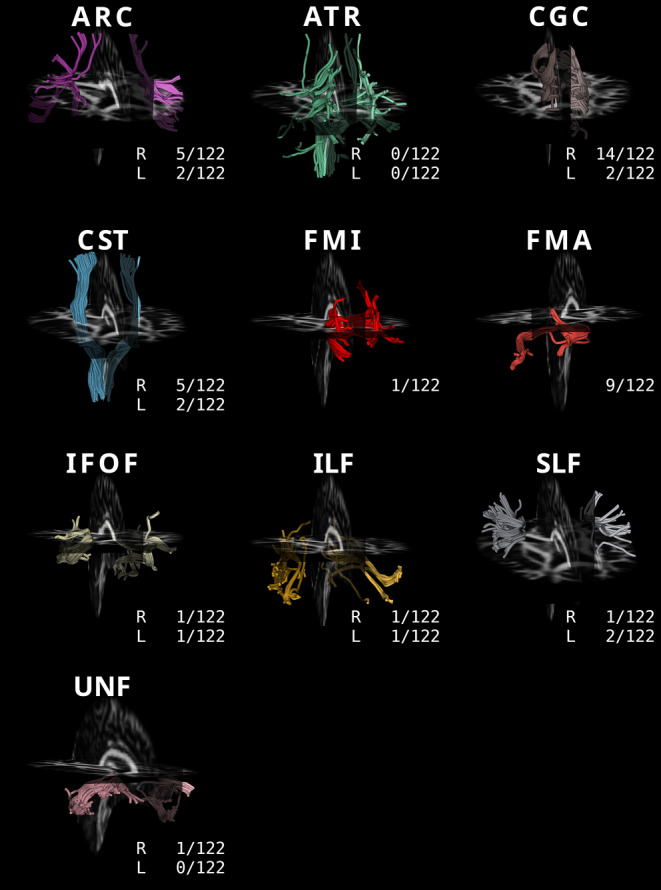
Morphology and anatomical positions of the 18 major fiber bundles. The number of subjects for each fiber that failed in the fiber tracking process is noted below each bundle. *ARC* arcuate fasciculus, *ATR* anterior thalamic radiation, *CGC* cingulum, *CST* corticospinal tract, *FMI* forceps minor, *FMA* forceps major, *IFOF* inferior fronto-occipital fasciculus, *ILF* inferior longitudinal fasciculus, *SLF* superior longitudinal fasciculus, *UNF* uncinate fasciculus, *R* right, *L* left

Comparing the T2DM and HC groups, there were no identifiable differences in mean diffusion metrics between the T2DM and HC groups across 18 fiber tracts after FDR correction (Table 2). Detailed information about the AFQ means of the diffusion metrics is listed in Table 3. Tract profile comparisons of the diffusion metrics between participants in the HC and T2DM groups were performed using AFQ to identify pointwise significant differences. The morphology and anatomical positions of the 18 major fiber tracts and the total number of subjects in which the tracking process failed are depicted in Fig. 2. Except for the fiber bundles described in the following sections, which showed differences between groups, none of the remaining fibers showed significant difference in the diffusion values.

**Table 2:** Mean diffusion metrics of 18 fiber tracts for the two groups

	FA		*P*	*P*_FDR_	AD		*P*	*P*_FDR_	MD		*P*	*P*_FDR_	RD		*P*	*P*_FDR_	ODI		*P*	*P*_FDR_	ICVF		*P*	*P*_FDR_
	T2DM	Healthy			T2DM	Healthy			T2DM	Healthy			T2DM	Healthy			T2DM	Healthy			T2DM	Healthy		
*ACR L*	0.54114 (0.02303)	0.54255 (0.02444)	0.593	0.864	0.88447 (0.02598)	0.88819 (0.02832)	0.370	0.864	0.00056 (0.00002)	0.00056 (0.00002)	0.474	0.864	0.39132 (0.01996)	0.39267 (0.01895)	0.835	0.939	0.21599 (0.01506)	0.21340 (0.01831)	0.294	0.864	0.59178 (0.02804)	0.59229 (0.02590)	0.826	0.939
*ACR R*	0.51632 (0.03228)	0.52466 (0.02949)	0.120	0.864	0.86944 (0.02591)	0.87713 (0.03027)	0.160	0.864	0.00056 (0.00002)	0.00056 (0.00002)	0.694	0.903	0.39937 (0.02630)	0.39415 (0.01981)	0.173	0.864	0.23464 (0.02160)	0.22788 (0.02284)	0.095	0.864	0.58465 (0.03190)	0.59082 (0.02773)	0.200	0.864
*ATR L*	0.46711 (0.01776)	0.46396 (0.02457)	0.694	0.903	0.87591 (0.02595)	0.88004 (0.02388)	0.370	0.864	0.00059 (0.00002)	0.00059 (0.00002)	0.425	0.864	0.43889 (0.01937)	0.44342 (0.02195)	0.400	0.864	0.26147 (0.01242)	0.26099 (0.01490)	0.658	0.883	0.56351 (0.02518)	0.56102 (0.06174)	0.999	0.999
*ATR R*	0.46276 (0.02615)	0.46399 (0.02133)	0.626	0.872	0.87590 (0.02929)	0.88242 (0.02411)	0.211	0.864	0.00059 (0.00002)	0.00059 (0.00002)	0.320	0.864	0.44166 (0.02345)	0.44518 (0.02153)	0.530	0.864	0.26217 (0.01714)	0.26085 (0.01323)	0.564	0.864	0.55323 (0.03115)	0.55758 (0.06086)	0.420	0.864
*CGC L*	0.52826 (0.03032)	0.52087 (0.03518)	0.337	0.864	0.90502 (0.02738)	0.90368 (0.02778)	0.935	0.994	0.00059 (0.00002)	0.00060 (0.00002)	0.480	0.864	0.42530 (0.02403)	0.42987 (0.02499)	0.470	0.864	0.22370 (0.01789)	0.22804 (0.02225)	0.393	0.864	0.53839 (0.02732)	0.53794 (0.03209)	0.898	0.970
*CGC R*	0.50262 (0.03418)	0.50250 (0.02710)	0.821	0.939	0.87508 (0.02588)	0.87746 (0.02793)	0.662	0.883	0.00059 (0.00002)	0.00059 (0.00002)	0.772	0.906	0.43595 (0.02573)	0.43740 (0.02055)	0.990	0.999	0.24126 (0.02334)	0.23949 (0.01997)	0.512	0.864	0.53105 (0.02969)	0.53215 (0.02605)	0.625	0.872
*CST L*	0.58675 (0.02517)	0.59020 (0.02608)	0.480	0.864	0.94661 (0.02850)	0.94994 (0.02441)	0.586	0.864	0.00055 (0.00001)	0.00055 (0.00001)	0.565	0.864	0.35379 (0.01769)	0.35056 (0.01867)	0.325	0.864	0.19013 (0.01645)	0.18658 (0.01573)	0.256	0.864	0.64457 (0.02177)	0.65310 (0.05010)	0.149	0.864
*CST R*	0.58353 (0.02181)	0.57852 (0.03051)	0.334	0.864	0.94927 (0.02570)	0.94827 (0.02912)	0.752	0.906	0.00056 (0.00001)	0.00056 (0.00002)	0.444	0.864	0.35908 (0.01609)	0.36237 (0.02441)	0.478	0.864	0.18930 (0.01484)	0.19026 (0.02147)	0.731	0.906	0.63325 (0.02058)	0.63666 (0.05415)	0.468	0.864
*FMI*	0.53970 (0.02888)	0.53578 (0.03430)	0.511	0.864	0.97959 (0.04006)	0.98182 (0.04790)	0.884	0.964	0.00060 (0.00002)	0.00061 (0.00002)	0.452	0.864	0.41210 (0.02597)	0.41648 (0.02908)	0.471	0.864	0.21839 (0.01669)	0.22098 (0.02257)	0.471	0.864	0.55061 (0.03060)	0.55695 (0.06296)	0.372	0.864
*FMA*	0.63930 (0.04299)	0.64679 (0.03725)	0.244	0.864	1.17356 (0.06267)	1.15875 (0.05831)	0.169	0.864	0.00066 (0.00005)	0.00064 (0.00004)	0.024*	0.648	0.38945 (0.05290)	0.37331 (0.04162)	0.038*	0.821	0.15897 (0.01984)	0.15735 (0.01807)	0.528	0.864	0.58109 (0.02840)	0.58884 (0.06197)	0.308	0.864
*IFOF L*	0.52619 (0.02981)	0.52560 (0.02211)	0.959	0.993	0.95752 (0.03532)	0.95870 (0.03122)	0.763	0.906	0.00062 (0.00002)	0.00062 (0.00002)	0.967	0.993	0.43079 (0.02540)	0.42931 (0.01735)	0.638	0.872	0.20827 (0.02019)	0.20636 (0.01548)	0.446	0.864	0.51461 (0.02718)	0.51676 (0.02054)	0.519	0.864
*IFOF R*	0.53175 (0.02351)	0.53210 (0.02292)	0.724	0.906	0.96074 (0.02884)	0.96204 (0.02928)	0.631	0.872	0.00062 (0.00002)	0.00062 (0.00002)	0.970	0.993	0.42968 (0.02126)	0.42838 (0.01895)	0.600	0.864	0.20472 (0.01457)	0.20327 (0.01517)	0.373	0.864	0.51515 (0.02509)	0.51702 (0.02262)	0.574	0.864
*ILF L*	0.50916 (0.02352)	0.50613 (0.02519)	0.496	0.864	0.96389 (0.03440)	0.95813 (0.03333)	0.375	0.864	0.00063 (0.00003)	0.00063 (0.00002)	0.427	0.864	0.45440 (0.02571)	0.45208 (0.02123)	0.567	0.864	0.22172 (0.01571)	0.22264 (0.01914)	0.804	0.934	0.49724 (0.02779)	0.49929 (0.02240)	0.582	0.864
*ILF R*	0.50913 (0.02232)	0.51024 (0.02156)	0.706	0.906	0.94635 (0.03141)	0.94949 (0.02237)	0.470	0.864	0.00062 (0.00002)	0.00062 (0.00002)	0.870	0.963	0.45031 (0.02161)	0.44793 (0.02016)	0.492	0.864	0.22645 (0.01488)	0.22297 (0.01064)	0.116	0.864	0.50188 (0.02558)	0.50531 (0.02438)	0.410	0.864
*SLF L*	0.49811 (0.02567)	0.50305 (0.02654)	0.242	0.864	0.85264 (0.02427)	0.85405 (0.02579)	0.583	0.864	0.00056 (0.00002)	0.00056 (0.00002)	0.769	0.906	0.41420 (0.02062)	0.41060 (0.02003)	0.332	0.864	0.24965 (0.01950)	0.24623 (0.02057)	0.267	0.864	0.58054 (0.02551)	0.58775 (0.02823)	0.166	0.864
*SLF R*	0.51150 (0.03078)	0.52296 (0.02901)	0.019*	0.648	0.86686 (0.02521)	0.87898 (0.02804)	0.008*	0.432	0.00056 (0.00002)	0.00056 (0.00002)	0.747	0.906	0.40707 (0.02338)	0.40255 (0.02209)	0.186	0.864	0.23702 (0.02409)	0.22537 (0.02050)	0.002*	0.216	0.58083 (0.02730)	0.58601 (0.02965)	0.257	0.864
*UNF L*	0.45556 (0.04363)	0.45556 (0.03697)	0.975	0.993	0.91962 (0.02461)	0.92442 (0.02520)	0.347	0.864	0.00063 (0.00003)	0.00063 (0.00002)	0.949	0.993	0.48261 (0.04020)	0.48201 (0.02946)	0.874	0.963	0.25670 (0.02570)	0.25516 (0.02446)	0.740	0.906	0.47613 (0.02863)	0.48422 (0.06569)	0.313	0.864
*UNF R*	0.47094 (0.02780)	0.46419 (0.03356)	0.337	0.864	0.93163 (0.02389)	0.93532 (0.03188)	0.546	0.864	0.00062 (0.00002)	0.00063 (0.00002)	0.202	0.864	0.46739 (0.02529)	0.47480 (0.02843)	0.215	0.864	0.24008 (0.01820)	0.24278 (0.02036)	0.512	0.864	0.48052 (0.02376)	0.48602 (0.06647)	0.436	0.864

*T2DM* type 2 diabetes mellitus, *HC* healthy controls, *FDR* false discovery rate, *ARC* arcuate fasciculus, *ATR* anterior thalamic radiation, *CGC* cingulum, *CST* corticospinal tract, *FMI* forceps minor, *FMA* forceps major, *IFOF* inferior fronto-occipital fasciculus, *ILF* inferior longitudinal fasciculus, *SLF* superior longitudinal fasciculus, *UNF* uncinate fasciculus, *R* right, *L* left

*indicates a significant difference between the two groups before false discovery rate correction at a significance level of 0.05

**Table 3:** Demographic and clinical information and cognitive measurements for participants in the two groups

Groups	T2DM	Healthy Control	*P* value
	(*n* = 61)	(*n* = 61)	
Age (years, mean ± SD)	47.57 ± 8.84	48.08 ± 10.86	0.777
Sex = male (%)	32 (52.5)	26 (42.6)	0.365
Education level (years, mean ± SD)	11.00 ± 4.03	10.97 ± 3.50	0.962
Systolic BP (mm Hg, mean ± SD)	129.20 ± 18.50	126.72 ± 17.80	0.453
Diastolic BP (mm Hg, mean ± SD)	84.43 ± 9.40	83.90 ± 11.23	0.780
BMI (mean ± SD)	23.91 ± 3.02	23.21 ± 3.26	0.216
Diabetes duration (years, mean ± SD)	4.06 ± 3.79	–	–
HbA1c (%, mean ± SD)	8.10 (7.00, 11.00)	–	–
HbA1c (mmol/mol, mean ± SD)	68 (53, 97)	–	–
FBG (mmol/L, mean ± SD)	8.78 ± 2.82	–	–
Fasting insulin (mU/L, mean ± SD)	12.51 ± 11.32	–	–
HOMA-IR (mean ± SD)	1.68 ± 1.23	–	–
MoCA (mean ± SD)	25.72 ± 3.24	25.95 ± 2.81	0.676
MMSE (median, 25%, 75% IQR)	28.50 (27.00, 29.75)	29.00 (28.00, 30.00)	0.191

Normally distributed data are expressed as the mean and standard deviation (*SD*) and as the median and 25% and 75% interquartile range (*IQR*) otherwise

*T2DM* type 2 diabetes mellitus, *HC* healthy controls, *BP* blood pressure, *BMI* body mass index, *HbA1c* glycosylated hemoglobin level, *FBG* fasting blood glucose, *FINS* fasting insulin level, *HOMA-IR* homeostatic model assessment for insulin resistance, *MoCA* Montreal Cognitive Assessment-Basic, *MMSE* Mini-Mental State Examination

#### Right SLF

**Fig. 3:**
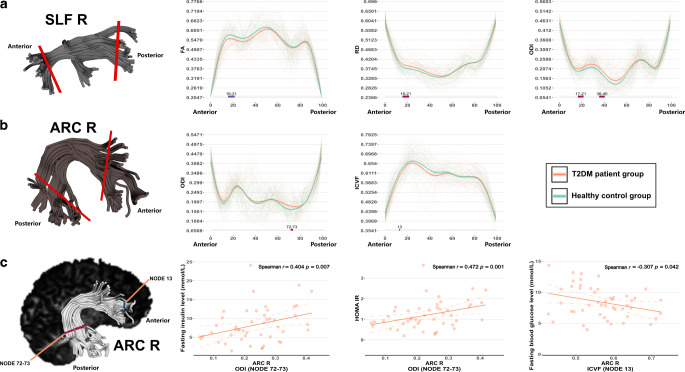
AFQ revealed differences in the right SLF (**a**), right ARC (**b**), and Spearman partial correlation analyses between aberrant ODI and ICVF values in the right ARC and fasting insulin, HOMA-IR, and fasting blood glucose levels in the T2DM group (**c**). The *gray shaded bars on the bottom of the plots* depict significantly altered locations on the fiber tracts, with *P*_FWE_ < 0.05; the *red colors* represent significantly increased metric values in the T2DM group compared to the HC group, and the *blue colors* represent significantly decreased metric values in the T2DM group compared to the HC group. *T2DM* type 2 diabetes mellitus, *HC* healthy controls, *FWE* familywise error, *AFQ* automatic fiber quantification, *SLF* superior longitudinal fasciculus, *ARC* arcuate fasciculus, *FA* fractional anisotropy, *RD* radial diffusivity, *ODI* orientation dispersion index, *ICVF* intracellular volume fraction, *R* right, *L* left

Compared with HC subjects, T2DM subjects demonstrated significant alterations in most diffusion metrics for the anterior part of the right SLF. Among these alterations, the FA was decreased and the RD increased in nodes 16-21, and the ODI increased in nodes 17-21 and nodes 36-40 (Fig. 3a).

#### Right ARC

Individuals with T2DM showed a significantly increased ODI in the posterior portion of the right ARC (nodes 72-73) and a decreased ICVF in the anterior part of the right ARC (node 13) (Fig. 3b).

#### Left ATR

**Fig. 4:**
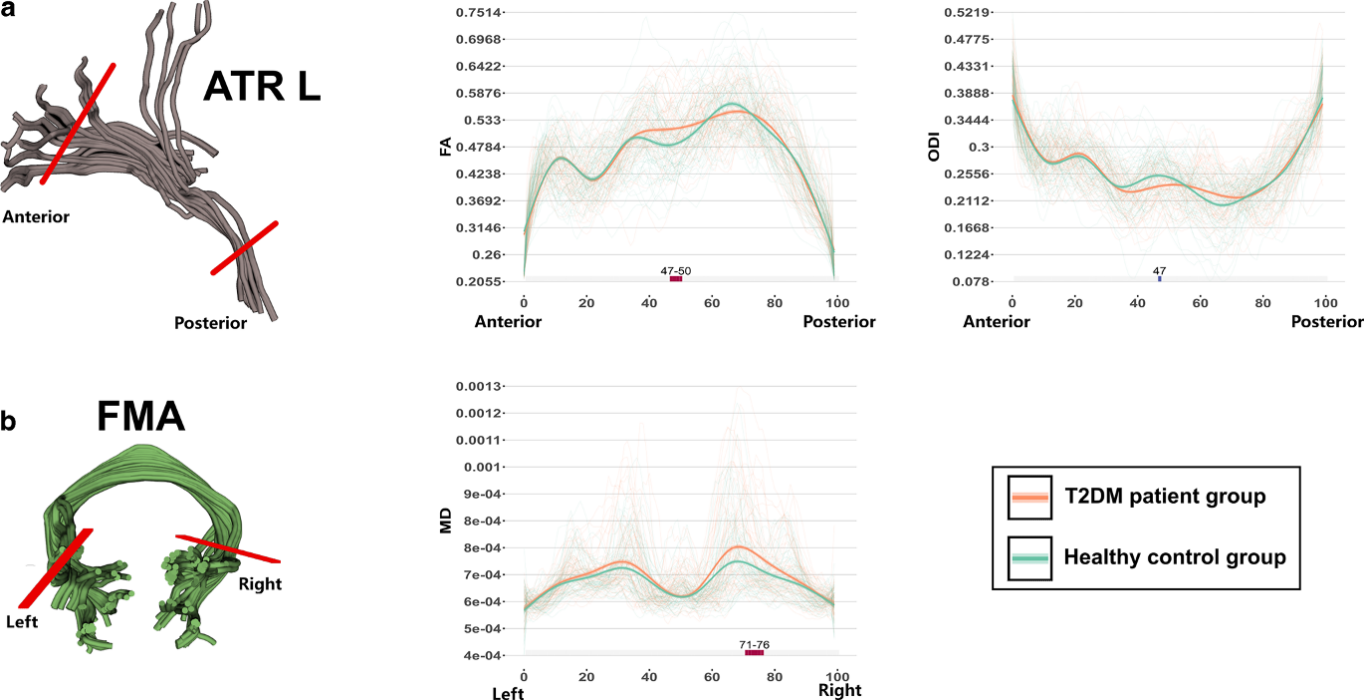
AFQ-revealed differences in the FMA (**a**) and left ATR (**b**). The *gray shaded bars on the bottom of the plots* depict significantly altered locations on the fiber tracts, with *P*_FWE_ < 0.05; the *red colors* represent significantly increased values in the T2DM group compared to the HC group, and the *blue colors* represent significantly decreased values in the T2DM group compared to the HC group. *T2DM* type 2 diabetes mellitus, *HC* healthy controls, *FWE* familywise error, *AFQ* automatic fiber quantification, *ATR* anterior thalamic radiation, *FMA* forceps major, *FA* fractional anisotropy, *ODI* orientation dispersion index, *MD* mean diffusivity, *L* left

The left ATR of individuals with T2DM exhibited significant differences that were mainly located in the middle part of the fiber bundle. Specifically, the FA (nodes 47-50) were significantly increased, and the ODI (node 47) was significantly decreased (Fig. 4a).

#### FMA

The FMA fibers of the individuals with T2DM showed significantly different MD values, mainly on the right posterior parts of the fiber bundle. The right posterior part of the tract demonstrated significantly increased MD in nodes 71-76 (Fig. 4b).

### Partial Correlation Analysis

**Fig. 5:**
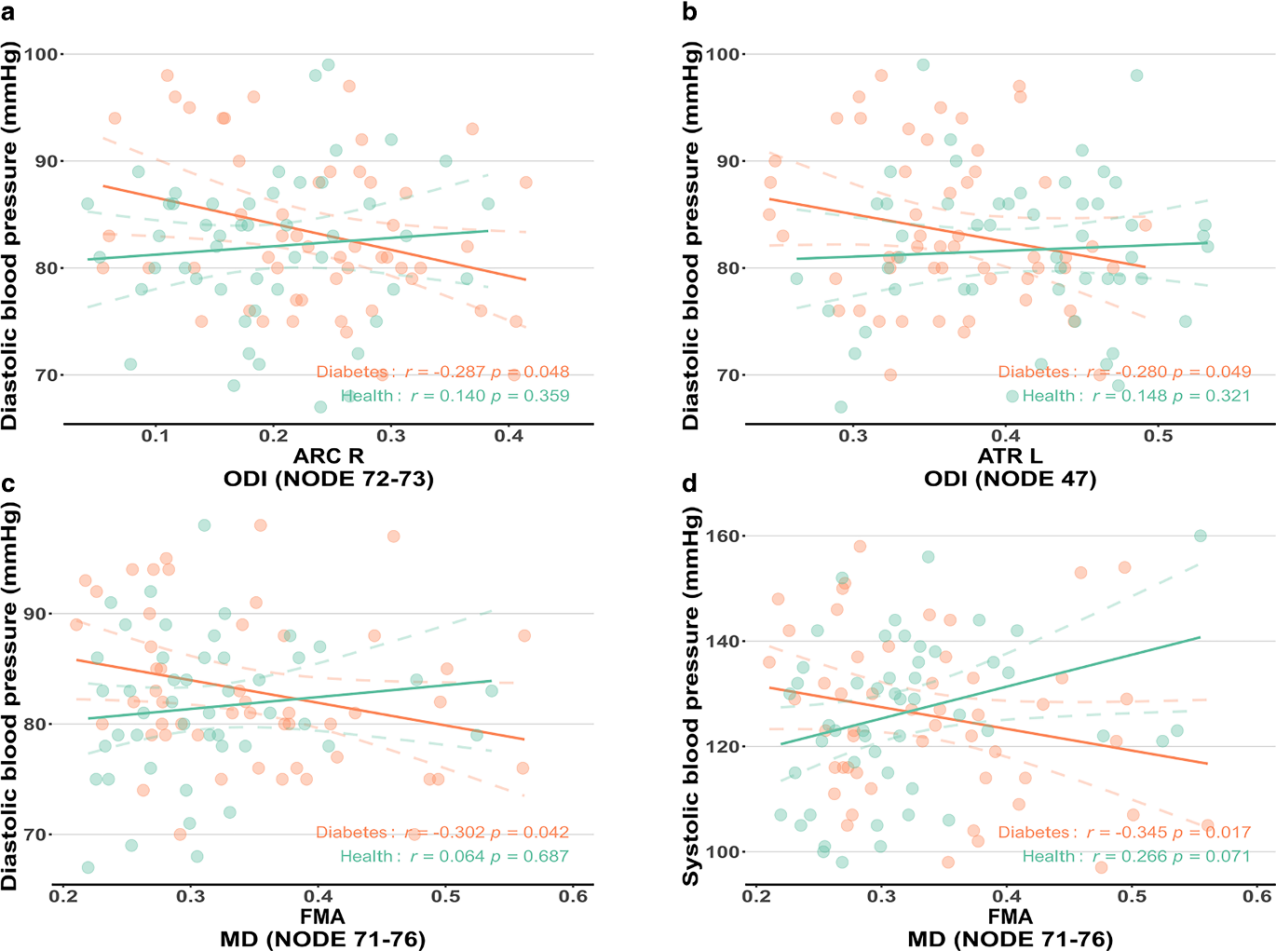
Spearman partial correlation analyses on the relationship of ODI values in the right ARC (nodes 72-72, **a**), left ATR (node 47, **b**) and diastolic blood pressure, and the relationship of the MD values in the FMA (nodes 71-76) and diastolic blood pressure (**c**) and systolic blood pressure (**d**). The *orange colors* represent the T2DM group; the *green colors* represent the HC group. *T2DM* type 2 diabetes mellitus, *HC* healthy controls, *ARC* arcuate fasciculus, *ATR* anterior thalamic radiation, *FMA* forceps major, *ODI* orientation dispersion index, *MD* mean diffusivity

Partial correlation analysis revealed that the ODI values in nodes 72–32 of the right ARC were positively correlated with the FINS level and HOMA-IR (Spearman *r* = 0.404, *P* = 0.007 and Spearman *r* = 0.472, *P* = 0.001, respectively) (Fig. 3c) and negatively correlated with DBP in T2DM patients (Spearman *r* = −0.287, *P* = 0.048, Fig. 5a). The ICVF values in node 13 of the right ARC were significantly associated with the FBG levels (Spearman *r* = −0.307, *P* = 0.042) (Fig. 3c). The ODI values in node 47 of the ATR were negatively correlated with DBP in the T2DM group (Spearman *r* = −0.280, *P* = 0.049, Fig. 5b). The MD values in nodes 71-76 of the FMA were negatively associated with DBP and SBP in T2DM patients (Spearman *r* = −0.345, *P* = 0.017 and Spearman *r* = −0.302, *P* = 0.042, respectively) (Fig. 5c, d). No significant findings were observed in the remaining partial correlation analyses.

## Discussion

T2DM is a common disease that affects the WM region of the brain [25, 26]. The WM contains fiber bundles that form important bridges for connecting grey matter (GM) structures and transmitting information between brain regions. The destruction of WM is thought to interrupt cortical–cortical or subcortical pathways related to certain important cognitive functions; i.e., the “disconnection hypothesis” plays a role in cognitive impairment [27]. Studies have demonstrated WM pathologies in T2DM patients through TBSS [28] and structural connectivity [29] perspectives, but the abnormalities in specific parts along the fiber tracts are still unclear. The main purpose of the present study was to identify the regions within the fiber tracts that are prone to T2DM-related brain damage.

Using TBSS, we first investigated the white matter characteristics of T2DM patients and healthy subjects. We found that the FA values were significantly reduced in T2DM patients compared with HCs in several WM regions, including the bilateral ATR, CST, IFOF, right SLF, left UNF, and FMI, while significantly decreased AD was observed in the left CST, FMA, and FMI regions. These findings are similar to those of previous studies, indicating that the wide range of microstructural damage observed by DTI is reliable across different diffusion imaging models [7, 30, 31]. We also detected significant ODI increases in two relatively small regions located in the right ATR, the CST, and the left IFOF, the ILF.

The FA is the most widely used DTI metric and indicates various characteristics of WM changes. A decreased FA may indicate a lower size and number of fibers and a decrease in the density of crossing fibers, as well as axonal degeneration, gliosis, and demyelination [32, 33]. It has been suggested that AD is sensitive to axonal pathologies [34]. Decreases in the AD are possibly due to increased debris from membrane disruption during axonal degeneration [35] or complex changes in fiber architecture [36]. Although the changes in the AD may not be sufficient to cause significant RD and MD differences, they may contribute to the decrease in FA in overlapping WM regions in T2DM patients. Some regions also displayed increased ODI in T2DM, which specifically refers to the aberrant directional disturbance of axons, meaning crossing, kissing, bending, or fanning fibers [13, 37] or axonal degeneration processes [47] in the overlapped regions in T2DM patients. Furthermore, some abnormal regions and tracts (e.g., CST, IFOF, FMA, and FMI) found in T2DM patients are similar to those observed in Alzheimer’s disease patients [38, 39], suggesting that similar neurodegenerative processes may underlie T2DM and Alzheimer’s disease [40, 41].

One study reported that patients with mild cognitive impairment and T2DM (decreased MoCA and MMSE scores) were characterized by a significantly lower ICVF in the temporal lobe than normal cognition healthy subjects, suggesting neurite density loss in associated brain regions in T2DM patients with cognitive deficits [42]; however, ICVF abnormalities were identified in the TBSS analysis. Although some participants showed reduced MoCA scores (< 26), all the participants had MMSE scores higher than 25. These findings were insufficient for defining cognitive dysfunction in our study; hence, we did not classify the subgroups according to patient cognitive status, which may have led to inconsistencies from previous results. Other studies [43, 44] have suggested that the MMSE-MoCA relationship may be different across different clinical situations. A diagnosis of cognitive impairment might be established with further investigations and comprehensive assessments after a suspicious MoCA or MMSE score in different diseases [45]. Axonal density decreases or other types of WM tract pathology in T2DM subjects may require further investigation based on more rigorous diagnostic criteria for cognitive impairment.

The AFQ results in the current study are mostly in line with the outcomes of TBSS, and AFQ localized abnormalities on fiber tracts more precisely and intuitively than TBSS. We found decreased FA in the anterior portion of the right SLF, which was similar to the results of TBSS. In contrast to the TBSS findings, AFQ analysis also showed increased RD and ODI values in the anterior portion of the right SLF. The T2DM patients exhibited higher ODI and lower ICVF values in the posterior and anterior portions of the right ARC, respectively, than the HC group. The SLF connects the frontal cortex to the occipital and parietal cortex and plays a major role in motor tasks, speech and language, and the default network. Damage to this tract can cause speech impairments such as anarthria and dysarthria [46]. ARC, which is considered to be a part of the SLF in some classification systems, also plays a major role in speech processing [47] and spatial processing in the right nondominant hemisphere [48]. Previous studies have found that the volume of frontal and parietal gray matter in T2DM patients is reduced [49], which emphasizes SLF and ARC injuries and their importance in T2DM brain damage [6, 7]. The AFQ results specifically showed abnormalities in the anterior part of the SLF, suggesting that the anterior portion of the SLF is vulnerable in T2DM. The decreased FA and increased RD further suggest axonal degeneration or demyelination processes in the right SLF, and the increased ODI indicates dispersion in neurite orientation and its trend to be lower in demyelinated lesions [50] and higher in the axonal degeneration process [51]. Thus, axonal degeneration may be the core feature of T2DM brain damage in the right SLF. Nonetheless, further studies using MRI sequences that are sensitive to myelin, such as magnetization transfer or myelin water imaging, would be necessary to confirm the findings.

ATR, a major fiber in the frontothalamic circuitry, is considered to be an important structural pathway for cognition and behavior [52]. A previous study showed that the functional connectivity between the thalamus and multiple cortical regions decreased in T2DM patients and highlighted the association with impairment of verbal fluency and working memory [53]. Surprisingly, the AFQ results conflicted with the TBSS findings that showed that the FA values of the anterior part of the left ATR increased, and the ODI values decreased in a similar portion, which indicates that axonal integrity increases. WM fiber integrity disturbance is frequently reported in various diseases, including T2DM, and FA decreases as a result [28]; however, NODDI-derived metrics may represent more specific histopathological damage [50], such as tau pathology, which may increase FA and decrease ODI values [14]. Our findings may suggest potential nontissue sources of damage to this fiber.

In AFQ analysis, we also found that MD increased in the right FMA in T2DM patients. The FMA is a white matter fiber bundle that connects the occipital lobes and crosses the midline via the splenium of the corpus callosum; damage to this bundle may affect occipital lobe function and lead to visual processing dysfunction [54]. Very few studies have reported microstructural alterations in the FMA in T2DM patients, and one study showed that obese T2DM patients had a lower AD in FMA regions than lean subjects [55]. Functional and macrostructural MRI studies have found that localized functional connectivity and gray matter volume decreased in the FMA-associated occipital regions in T2DM patients [56, 57]. The abnormally increased MD values in FMA indicate higher mobility of water molecules, which may suggest pathologies including edema, axonal degeneration, or demyelination [58]. Given the limited information that a single altered diffusion metric can provide, the specific microstructural pathology of FMA in T2DM patients may warrant further investigation.

The correlation analysis for ARC revealed that the ODI values of nodes 72-73 were positively correlated with FINS and HOMA-IR in T2DM patients, and the ICVF values of node 13 were negatively associated with FBG, which suggests that the insulin level and insulin resistance may be a factor in the dispersion of the axonal orientation and that glucose status may be associated with the axonal density of the right ARC. Various studies have linked the relationship between diabetic status and central nervous damage [2, 42, 59]. Our correlation analysis indicated that diabetes statuses, such as glucose control, insulin levels, and insulin resistance, may be potential factors affecting T2DM brain damage to the right ARC. The correlation analysis also showed some interesting findings regarding the relationship between blood pressure and fiber tract abnormalities. The DBP levels were negatively correlated with the values in nodes 72-73 of the right ARC in the T2DM group but not in the HC group. On the other hand, there was a negative relationship between the altered node ODI values of the left ATR and DBP. Similar to the relationship between the right ARC, left ATR, and DBP in our findings, correlation analysis revealed that the SBP and DBP were negatively correlated with the MD values in nodes 71-76 of the FMA. No such relationships were observed in the HC group. It is still difficult to conclude whether the phenomena were caused by regional compensatory mechanisms to the hemodynamic changes. Although the deleterious effects of hypertension on the brain have been frequently reported, compensatory neural changes have also been reported [60, 61]. Additionally, we included mild or moderate hypertensive subjects, and the association between WM tract abnormalities and severe hypertension was not studied in the current study. Further studies on cerebral hemodynamics and the associated microstructural histopathological alterations are needed to elucidate the role of blood pressure in T2DM brain damage.

In the current study, we adopted an advanced DSI protocol to acquire our DWI data and reconstructed the DSI data with DTI and NODDI models. Conventional DTI analysis is often susceptible to the partial volume effect of cerebrospinal fluid (CSF), which leads to deviations in the results [62]. The NODDI model based on the multi-interval biophysical model can effectively separate the CSF part of the diffuse signal, thus precluding the influence of the partial volume effect caused by the CSF [63, 64]. The NODDI model also provides sensitive estimates of the density and directional dispersion of neural processes isolating the two key factors of FA and is an advanced model for analyzing microstructures in fibers [65]. In this procedure, each voxel is divided into three microstructure intervals that provide corresponding indicators: (1) the ICVF, which reflects the density of neurites (axons or dendrites); (2) the ODI, which reflects the degree of neurite coherence and (3) the isotropic volume fraction, which reflects the proportion of free water (i.e., CSF) in a voxel [65]. NODDI modalities offer greater sensitivity in the detection of cortical abnormalities than traditional MR modalities in neurodegenerative disorders, such as Parkinson’s disease [66]. The NODDI model can be fitted even with traditional single-shell DWI data; however, the high angular nature of *q*-space samplings, such as the DSI we used in the current study, is advantageous for more accurately capturing the NODDI features of neurite orientation dispersion and density change [15], and our results also indicated that together with NODDI and DTI, more accurate interpretations about the microstructural alterations of the fibers underlying T2DM brain damage can be obtained. Nonetheless, the NODDI model has been implemented in various studies on neurodegeneration and psychiatric diseases [65], and the application of the NODDI model in T2DM still warrants further investigation.

The diffusion characteristics of WM may change along each tract, which varies individually. We performed TBSS and compared the mean diffusion metrics along the fiber tracts, and the results revealed that TBSS is still a reliable method for WM analysis, whereby the mean diffusion metrics showed no significant differences in the two groups; however, voxel-based techniques, such as TBSS, cannot provide the location-specific properties of WM integrity in subjects’ fiber tracts, which vary in shape [67]. Similar to regions of interest analysis or traditional tractography, which average the diffusion metrics along the entire length of the tracts, the mean diffusion metric comparisons may be more suitable for hypothesis-driven or connectome studies since significant *P* values would be correct for multiple comparisons that mask the findings. Hence, to further investigate and quantify T2DM alterations to specific portions of fibers, we used a new emerging AFQ technique, which was first proposed by Yeatman et al. [16], implemented in the MATLAB platform and recently migrated to Python [68]. AFQ is a fully automated method that can be used to effectively track the main WM fiber bundles at the individual level and analyze the diffusion characteristics in an anatomically equivalent position along their trajectory [16]. Our results suggest that AFQ is a sensitive approach for detecting the specific type of pathology in a small portion of WM fiber tracts and may provide new insights into WM degeneration in T2DM patients.

There are several limitations in the present cross-sectional study. First, we included relatively early phase (diabetes duration of 4.06 years on average) T2DM patients younger than 60 years old. It has been reported that a longer duration of diabetes is associated with brain volume loss and increased dementia rates after age 70 years [69]. Longitudinal follow-up is needed to elucidate the effects of diabetes duration on the brain microstructure and its dynamic patterns. Second, we only studied relationships between the neuroimaging findings and the general cognitive performances (MoCA, MMSE) as well as other clinical tests in the current study. Psychological and neurological tests on specific cognition domains can be further investigated to assess the structural and associated cognitive functional changes. Third, we identified some abnormal fiber bundles, but subdivisions of some major bundles were not studied in the present study. For example, the SLF can be further divided into three major branches that have distinct functions in normal cognition processes. Applying AFQ to the subdivisions of major bundles may provide informative insights into neurodegeneration. Fifth, we measured fasting fingertip blood glucose but not fasting plasma glucose in HC subjects and thus may not have accurately reflected their fasting glucose status. Finally, we used the DTI model and determinant tracking algorithm in AFQ. More advanced algorithms are available for DSI data processing and fiber tracking, which truly take advantage of the DSI, allowing the delineation of fibers or fiber pathways that have more chiasmata and a complex architecture of neural pathways [70, 71].

## Conclusion

The AFQ is a promising analytical approach for more precise localization of WM tract damage in T2DM patients. Better understanding of the specific types and portions of WM tract abnormalities can be achieved together with DTI and NODDI. The anterior part of the right SLF is prone to axonal degeneration in T2DM. Other kinds of microstructural alterations in the right ARC, left ATR and FMA can also be accurately identified. These microstructural alterations may be associated with insulin resistance status and glucose and blood pressure control in T2DM patients. The microstructural findings may provide neuroimaging evidence of brain damage in T2DM patients.

## Supplementary Information

**Table S1.** The microstructural abnormalities of white matter tracts reflected by FA in T2DM patients (T2DM < HC)

**Table S2.** The microstructural abnormalities of white matter tracts are reflected by AD in T2DM patients (T2DM < HC)

**Table S3.** The microstructural abnormalities of white matter tracts are reflected by ODI in T2DM patients (T2DM > HC)

## Abbreviations

AD: Axial diffusivity
AFQ: Automatic fiber quantification
ARC: Arcuate fasciculus
ATR: Anterior thalamic radiation
BMI: Body mass index
CGC: Cingulum
CSF: Cerebrospinal fluid
CST: Corticospinal tract
DBP: Diastolic blood pressure
DSI: Diffusion spectrum imaging
DTI: Diffusion tensor imaging
DWI: Diffusion weighted imaging
FA: Fractional anisotropy
FBG: Fasting blood glucose
FDR: False discovery rate
FINS: Fasting insulin
FMA: Forceps major
FMI: Forceps minor
FWE: Familywise error
HOMA-IR: Homeostatic model assessment for insulin resistance
ICVF: Intracellular volume fraction
IFOF: Inferior fronto-occipital fasciculus
ILF: Inferior longitudinal fasciculus
MD: Mean diffusivity
ODI: Orientation dispersion index
QC: Quality control
RD: Radial diffusivity
SBP: Systolic blood pressure
SLF: Superior longitudinal fasciculus
TBSS: Tract-based spatial statistics
UNF: Uncinate fasciculus
WM: White matter
