# Supplementary material for: White Matter Characteristics of Damage Along Fiber Tracts in Patients with Type 2 Diabetes Mellitus
Source: Clin Neuroradiol. 2022 Sep 16;33(2):327–41. doi: 10.1007/s00062-022-01213-7 (PMC10220145; doi:10.1007/s00062-022-01213-7)
Supplement: Supplementary file 1 — Table S1. The microstructural abnormalities of white matter tracts reflected by FA in T2DM patients (T2DM < HC) [file 62_2022_1213_MOESM1_ESM.docx]

**Table S1.** The microstructural abnormalities of white matter tracts reflected by FA in T2DM patients (T2DM < HC).

| Cluster Index | Voxels | *p* | MNI coordinates of the peak voxel | | | Side | Anatomical region |
| --- | --- | --- | --- | --- | --- | --- | --- |
|  |  |  | X | Y | Z |  |  |
| 1 | 4520 | 0.03 | 8 | -8 | -13 | Right | Anterior thalamic radiation |
|  |  |  |  |  |  |  | Corticospinal tract |
|  |  |  |  |  |  |  | Inferior fronto-occipital  fasciculus |
|  |  |  |  |  |  | - | Forceps minor |
|  | | | | | | | |
| 2 | 2337 | 0.036 | -18 | -18 | 36 | Left | Corticospinal tract |
|  |  |  |  |  |  | - | Forceps minor |
|  | | | | | | | |
| 3 | 1363 | 0.038 | 7 | -38 | -32 | Left | Anterior thalamic radiation |
|  |  |  |  |  |  | Right | Anterior thalamic radiation |
|  | | | | | | | |
| 4 | 652 | 0.037 | -18 | -19 | -7 | Left | Anterior thalamic radiation |
|  |  |  |  |  |  |  | Corticospinal tract |
|  | | | | | | | |
| 5 | 369 | 0.04 | -8 | 0 | -15 | Left | Anterior thalamic radiation |
|  | | | | | | | |
| 6 | 363 | 0.045 | -21 | 14 | 10 | Left | Anterior thalamic radiation |
|  |  |  |  |  |  |  | Inferior fronto-occipital  fasciculus |
|  |  |  |  |  |  |  | Uncinate fasciculus |
|  | | | | | | | |
| 7 | 261 | 0.046 | 33 | -12 | 42 | Right | Superior longitudinal  fasciculus |
|  |  |  |  |  |  |  | Superior longitudinal  fasciculus (temporal part) |
|  | | | | | | | |
| 8 | 257 | 0.046 | 20 | -42 | 28 | Right | Inferior fronto-occipital  fasciculus |
|  |  |  |  |  |  |  | Superior longitudinal  fasciculus |
|  | | | | | | | |
| 9 | 71 | 0.047 | 37 | 34 | 10 | Right | Inferior fronto-occipital  fasciculus |
|  |  |  |  |  |  |  | Anterior thalamic radiation |

FA, diffusion tensor imaging-fractional anisotropy; T2DM, type 2 diabetes mellitus; HC, healthy control.
