# Supplementary material for: White Matter Characteristics of Damage Along Fiber Tracts in Patients with Type 2 Diabetes Mellitus
Source: Clin Neuroradiol. 2022 Sep 16;33(2):327–41. doi: 10.1007/s00062-022-01213-7 (PMC10220145; doi:10.1007/s00062-022-01213-7)
Supplement: Supplementary file 2 — Table S2. The microstructural abnormalities of white matter tracts are reflected by AD in T2DM patients (T2DM < HC) [file 62_2022_1213_MOESM2_ESM.docx]

**Table S2.** The microstructural abnormalities of white matter tracts are reflected by AD in T2DM patients (T2DM < HC).

| Cluster Index | Voxels | *p* | MNI coordinates of the peak voxel | | | Side | Anatomical region |
| --- | --- | --- | --- | --- | --- | --- | --- |
|  |  |  | X | Y | Z |  |  |
| 1 | 1847 | 0.034 | -18 | -50 | 18 | Left | Corticospinal tract |
|  |  |  |  |  |  | - | Forceps major |
|  |  |  |  |  |  | - | Forceps minor |
|  | | | | | | | |
| 2 | 680 | 0.032 | 18 | -42 | 27 | - | Forceps major |

AD, diffusion tensor imaging- axial diffusivity; T2DM, type 2 diabetes mellitus; HC, healthy control.
