# Supplementary material for: White Matter Characteristics of Damage Along Fiber Tracts in Patients with Type 2 Diabetes Mellitus
Source: Clin Neuroradiol. 2022 Sep 16;33(2):327–41. doi: 10.1007/s00062-022-01213-7 (PMC10220145; doi:10.1007/s00062-022-01213-7)
Supplement: Supplementary file 3 — Table S3. The microstructural abnormalities of white matter tracts are reflected by ODI in T2DM patients (T2DM > HC) [file 62_2022_1213_MOESM3_ESM.docx]

**Table S3.** The microstructural abnormalities of white matter tracts are reflected by ODI in T2DM patients (T2DM > HC).

| Cluster Index | Voxels | *p* | MNI coordinates of the peak voxel | | | Side | Anatomical region |
| --- | --- | --- | --- | --- | --- | --- | --- |
|  |  |  | X | Y | Z |  |  |
| 1 | 31 | 0.039 | 7 | -38 | -32 | Right | Anterior thalamic radiation |
|  |  |  |  |  |  |  | Corticospinal tract |
|  | | | | | | | |
| 2 | 16 | 0.047 | -30 | -37 | 18 | Left | Inferior fronto-occipital  fasciculus |
|  |  |  |  |  |  |  | Inferior longitudinal  fasciculus |

ODI, orientation dispersion index ; T2DM, type 2 diabetes mellitus; HC, healthy control.
